# Cognitive Profiles of Amyotrophic Lateral Sclerosis Differ in Resting-State Functional Connectivity: An fMRI Study

**DOI:** 10.3389/fnins.2021.682100

**Published:** 2021-06-23

**Authors:** Anna G. M. Temp, Martin Dyrba, Charlotte Büttner, Elisabeth Kasper, Judith Machts, Jörn Kaufmann, Stefan Vielhaber, Stefan Teipel, Johannes Prudlo

**Affiliations:** ^1^German Center for Neurodegenerative Diseases (DZNE), Rostock, Germany; ^2^Department of Neurology, Rostock University Medical Center, Rostock, Germany; ^3^German Center for Neurodegenerative Diseases (DZNE), Magdeburg, Germany; ^4^Department of Neurology, Otto-von-Guericke University Magdeburg, Magdeburg, Germany; ^5^Department of Psychosomatic Medicine, Rostock University Medical Center, Rostock, Germany

**Keywords:** amyotrophic lateral sclerosis (ALS), resting-state functional magnetic resonance imaging, cognition, cognitive dysfunction, frontotemporal lobar degeneration

## Abstract

**Background:**

Half of all amyotrophic lateral sclerosis-frontotemporal spectrum disorder (ALS-FTSD) patients are classified as cognitively impaired, of which 10% have frontotemporal dementia (FTD), and an additional 40% suffer from a frontotemporal syndrome not severe enough to be described as dementia (cognitively impaired/ALSci). As changes in cerebral function measured by resting-state magnet resonance imaging (rs-fMRI) are known in ALS, we investigated whether group differences in resting-state functional connectivity (RSFC) networks could be observed between ALS patients with different cognitive profiles against healthy controls (HC). Furthermore, we correlated cognition and motor functioning with network connectivity.

**Methods:**

Healthy controls, 69, and 97 ALS patients underwent functional MRI scanning and cognitive assessment. The ALS patients were categorized as non-impaired (ALSni; *n* = 68), cognitively impaired (ALSci; *n* = 21), and ALS-FTD (*n* = 8). Group differences in connectivity of the default mode network (DMN), motor network (MN), and ventral attention network (VAN) were investigated using a full-factorial model; correlations between global cognitive performance, shifting, and motor symptom severity were established using Pearson’s correlation.

**Results:**

At a liberal alpha level of uncorrected *p* < 0.005 and a cluster size exceeding 20 voxels, we found widespread decreases in functional connectivity in all three networks when comparing ALS patients to HC. Similar patterns of hypoconnectivity in the bilateral motor cortices and frontotemporal emerged when comparing the ALSci and ALS-FTD patients to those not cognitively impaired. Hyperconnectivity in the DMN temporal gyrus correlated with worse global cognition; moreover, hyperconnectivity in the VAN thalamus, insula, and putamen correlated with worse shifting ability. Better-preserved motor function correlated with higher MN connectivity. Only the motor-related effects prevailed at a more conservative significance level of *p*_*FDR*_< 0.001.

**Conclusion:**

Resting-state functional connectivity differs between cognitive profiles of ALS and is directly associated with clinical presentation, specifically with motor function, and cognitive shifting.

## Introduction

Cognitive impairment falling short of dementia occurs in approximately 35% of patients with amyotrophic lateral sclerosis (ALS), and a further 10% develop a frontotemporal dementia (Rippon et al., 2006; Montuschi et al., 2015). Cognitive impairment in non-demented ALS patients characteristically includes executive and language deficits (Beeldman et al., 2016). The revised consensus criteria for the diagnosis of ALS propose a classification scheme based on deficits in specific cognitive domains (Strong et al., 2017): patients are categorized as non-impaired (ALSni), cognitively impaired (ALSci), behaviorally impaired (ALSbi), cognitively and behaviorally impaired but not demented (ALScbi), or as having frontotemporal dementia (ALS-FTD).

Shen et al. (2018) reported group differences in frontotemporal cerebral blood flow between ALSni, ALSci, and ALS-FTD (Shen et al., 2018). Resting-state functional connectivity (RSFC) analyses based on the BOLD signal in fMRI have shown differences between healthy controls (HCs) and ALS patients in several neuronal networks. Schulthess et al. (2016) reported a general expansion (i.e., an increase in RSFC) of ALS RSFC maps in the default mode network (DMN), motor network (MN), and ventral attention network (VAN) over time. Additionally, they reported that the expansions of the MN followed a pattern similar to the TDP-43 distribution in the brain. Current evidence regarding network expansion or contraction is heterogeneous. In the DMN, hyperconnectivity was found in the left precuneus in ALS patients when compared with healthy controls (Agosta et al., 2013; Li et al., 2017). In contrast, hypoconnectivity in the DMN has been reported in the ventral anterior cingulate cortex, the posterior cingulate cortex (Mohammadi et al., 2009; Agosta et al., 2013; Li et al., 2017), the orbitofrontal cortex, and the medial prefrontal cortex (Mohammadi et al., 2009; Schulthess et al., 2016; Li et al., 2017). This is in line with the notion that ALS patients seem to show lower RSFC in the anterior- and higher connectivity in the posterior nodes of the DMN than HC (Li et al., 2017). A further distinction into ALS with bulbar onset and ALS with limb onset by Trojsi et al. (2017) showed an even stronger increase in posterior RSFC in patients with bulbar onset.

Given that ALS is considered a multi-systemic disorder, which includes cognitive and motor impairment, it is of interest to establish a possible association between clinical symptomatology and possibly underlying changes in RSFC. The aim of the present study was to investigate differences between ALSni, ALSci, and ALS-FTD in resting-state functional connectivity. We expected to replicate group differences between ALS patients compared with HC in the DMN, VAN, and MN (Schulthess et al., 2016). We further expected a decreased functional connectivity to correlate with worse cognitive and motor function.

## Materials and Methods

### Participants

We recruited 97 ALS patients and 69 HCs from the university hospitals and German Centre for Neurodegenerative Diseases (DZNE) sites in Magdeburg and Rostock, Germany. Exclusion criteria were a history of brain injury, epilepsy, or psychiatric illnesses. Healthy controls were screened for normal cognition using the Montreal Cognitive Assessment (MoCA) with a cutoff of 26/30 (Nasreddine et al., 2005). ALS-FTSD patients were diagnosed according to the revised El Escorial criteria (Brooks et al., 2000). Cognitive classification was undertaken in accordance with the Strong and Rascovsky criteria (Rascovsky et al., 2011; Strong et al., 2017). This included thorough neuropsychological testing [see Kasper, Schuster (Kasper et al., 2015)] and behavioral assessment with the *Frontal Systems Behavior Scale* (Grace and Malloy, 2001). All patients underwent genetic testing for the *C9orf72* repeat expansion. In the case of a positive family history, young onset, or juvenile ALS, additional genes (*SOD1* and *TARDBP*) were examined. The low prevalence of 2% for *SOD1* and *C9orf72* is congruent with that previously reported for Northern Germany (Krüger et al., 2016).

The HC mean and SD (Table 1) were used to standardize the patients’ performance into z-scores, with *z* ≤ −2 reflecting an impaired performance. Patients were classified as ALSci (*n* = 21) if they scored at least two standard deviations below the HCs’ mean in a verbal fluency test or in two non-overlapping executive tests (following Strong et al., 4). Patients who met the Rascovsky criteria for FTD (Rascovsky et al., 2011) were classified as ALS-FTD (*n* = 8). Sixty-eight patients were ALSni. Table 1 shows that compared with ALSni, the ALSci patients were older, had a lower verbal IQ, and worse cognition measured by the MoCa index. ALS patients with frontotemporal patients also had a lower verbal IQ, MoCa index, and worse shifting ability.

**TABLE 1:** Demographic background of the participants.

Variable	HC	ALSni	ALSci	ALS-FTD
N	69	68	21	8
Site (HRO/MD)	41/28	23/44	10/11	4/4
Sex (f/m)	28/41	24/44	4/17	4/4
Handedness (R/L/A)	62/5/2	63/4/0/1 missing	18/2/0/1 missing	7/0/0/1 missing
Age (years)	59.73 (10.92)	58.41 (9.88)	64.95 (11.73)*	65.00 (7.05)
Education (years)	13.64 (1.95)	13.36 (2.61)	12.19 (1.72)	12.75 (2.44)
Verbal IQ	106.10 (7.95)	102.68 (10.95)	94.00 (8.42)**	94.13 (14.02)*
Disease duration (months)	–	36.20 (45.75)	39.50 (63.09)	33.14 (36.75)
ALSFRS-R	–	37.74 (6.20)	38.14 (5.18)	39.50 (7.45)
MoCa Index		0.87 (0.08)	0.75 (0.12)***	0.66 (0.15)***
TMT B/A		2.39 (0.96)	2.75 (1.37)	3.42 (1.66)*
Onset Type (spinal/bulbar)		38/17/13 uncertain	12/5/4 uncertain	2/4/2 uncertain
Genetic variant				
SOD1		2	0	0
C9orf72		1	1	1
VAPB (ALS8)		1	0	0

*p < 0.05, **p < 0.01, ***p < 0.001 compared with ALSni.

*HC, healthy control; ALSni, amyotrophic lateral sclerosis patients categorized as non-impaired; ALSci, ALS patients cognitively impaired; MoCa index, Montreal Cognitive Assessment Index.*

All participants provided written informed consent. The study was conducted according to the Declaration of Helsinki and approved by the local medical ethics committee at each site.

### Measurements

#### Montreal Cognitive Assessment (MoCA)

This tool screens eight cognitive domains (visuospatial/executive, naming, memory, attention, language, abstraction, delayed recall, and orientation) using 10 tasks and has been shown to detect impairment reliably in ALS patients (Taule et al., 2020). The MoCA was chosen for the DMN because the DMN is not sensitive to specific cognitive tasks but has been shown to be associated with overall cognitive impairment in ALS and other neurodegenerative diseases (Rocca et al., 2010; Anticevic et al., 2012; Wang et al., 2013). The maximally obtainable number of points is 30. However, five of these points are potentially unobtainable by ALS patients due to motor impairment of the limbs or dysarthria (TMT-B, cube copy, clock drawing, letter A tapping, verbal fluency). Consequently, we calculated an index for each participant, based on his or her individual motor impairment. The index conveys the successfully obtained MoCA points as a percentage of the maximal points available to each participant, as limited by their motor impairment. Indices were standardized as z-scores to reflect global cognitive impairment.

#### Trail-Making Test, Ratio B/A

We chose the TMT for the VAN because the VAN typically reacts to stimulus-driven attentional control, visual search, and short-term memory tasks (Shulman et al., 2003, 2007; Wang et al., 2013; Vossel et al., 2014). The time taken to complete TMT-B was divided by the time taken to complete TMT-A to correct for the influence of motor impairment on the cognitive task of shifting within the executive domain. Standardized z-scores were used for analysis, to reflect impairment.

#### Verbal IQ

Verbal IQ was included because increased RSFC may be associated with higher levels of intelligence in healthy persons (Song et al., 2008). We estimated premorbid IQ after disease onset. As executive dysfunction is common in ALS and would diminish performance on classical IQ estimation tests, we opted for a vocabulary-based test (Schmidt and Metzler, 1992). We chose vocabulary as a proxy because it remains intact throughout the course of ALS (Neudert et al., 2001) and because this particular test has been shown to measure verbal-crystallized intelligence reliably (Schipolowski et al., 2014). Raw vocabulary scores were converted to an IQ estimate based on published norms (Schmidt and Metzler, 1992).

### MR Image Acquisition and Processing

Study procedures and scanning protocols had been prospectively harmonized between the two study sites. Each site was equipped with a 3T Siemens MAGNETOM Verio scanner with 32-channel phased array head coils (Siemens, Erlangen, Germany). Functional MRI was based on an echo-planar imaging sequence with a repetition time of 2,200 ms, echo time of 30 ms, flip angle of 80°, isotropic voxel size of 3.5 mm, 34 slices, acquisition matrix = 64 × 64 × 34, and an interleaved acquisition scheme starting with even slices. The sequence took 11 min and 4 s, yielding in total 300 volumes. During the functional scan, participants were asked to stay awake, relaxed, motionless, and keep their eyes closed. No visual or auditory stimuli were presented. In the same session, a high-resolution T1-weighted structural MRI scan was obtained using a 3D magnetization-prepared rapid acquisition with gradient echo (MPRAGE) sequence. Scanning parameters were an echo time of 4.82 ms, repetition time of 2,500 ms, inversion time of 1,100 ms, flip angle of 7°, isotropic voxel size of 1 mm, and matrix size of 256 × 256 × 192.

Image pre-processing was done using the Data Processing Assistant for Resting-State fMRI–Advanced (DPARSFA V4.3) (Chao-Gan and Yu-Feng, 2010). For functional imaging data, preprocessing included the removal of the first 10 volumes to ensure image gradient field stabilization, slice time correction, and realignment to the temporal mean image, and coregistration of the anatomical T1 weighted image to the mean functional image. Functional data was cleaned for nuisance covariates (white matter and CSF signal, head motion) and frequency filtered by a bandpass filter in the range 0.01 and 0.1 Hz. T1 images were then segmented and normalized to a study-specific template in MNI-alike reference space using the Diffeomorphic Anatomical Registration Through Exponentiated Lie algebra (DARTEL) algorithm. The resulting deformation fields were applied to the functional scans to warp them to the MNI-alike reference space. Finally, functional images were smoothed using a 6-mm full-width half-maximum (FWHM) isotropic Gaussian kernel. Following Schulthess et al. (2016), functional connectivity maps were calculated by placing a spherical seed with a 5-mm radius at the following (x y z) MNI positions: MN voxel-seed in motor cortex (−25 −37 65), DMN seed in posterior cingulate cortex (0 −55 26), and VAN seed in the ventral striatum (−11 13 0). As the functional connectivity metric is defined as the Pearson correlation coefficient between the seed region’s time series and all other voxels’ time series, Fisher’s Z transformation was applied to adjust the correlation coefficients to be normally distributed, as required by the subsequent statistical analysis.

### Statistical Analysis

Statistical analyses were performed in Statistical Parametric Mapping (SPM12, release 5236, Wellcome Centre for Human Neuroimaging, London, United Kingdom). Prior to our main analyses, we inspected the normal distribution and homogeneity of variance in functional connectivity across groups and networks. In preparation for the main analyses, we performed one-sample *t*-tests in each functional network of the HC to restrict the subsequent analyses to these specific functional network regions. Mean functional connectivity maps were thresholded at *r* > 0.7 to generate the network masks, which were then applied in the main analyses, one full-factorial design per network. First, we compared group differences in network connectivity. We compared HC with all ALS patient groups combined before proceeding with specified contrasts. These included the expectation that HCs’ functional connectivity would be higher than those of all patient groups in all networks, and that ALSni patients’ functional connectivity would be higher than those of ALSci and ALS-FTD patients in all networks. We further specified a contrast with the expectation that ALSci patients’ functional connectivity would be higher than that of ALS-FTD patients. Second, we conducted three multiple regressions between functional connectivity and clinical presentation in the ALS groups. We correlated connectivity strength of the DMN with standardized MoCA index scores, connectivity strength of the MN with ALSFRS-R raw score, and connectivity strength of the VAN with standardized TMT ratio scores. Given that first, higher RSFC may be associated with higher levels of intelligence (Song et al., 2008), and second, group differences in verbal-crystallized intelligence emerged in our participants (see Table 1), we included verbal IQ as a covariate. All analyses were corrected for age, sex, and verbal IQ. We applied a significance threshold of *p* < 0.001, with a false detection rate (FDR) error correction for multiple comparisons, and a cluster size threshold of *k* ≥ 20. As effect sizes are considered to be the most important outcome of any empirical study (Sullivan and Feinn, 2012; Lakens, 2013), we converted t values into Cohen’s d effect sizes standardized on between-group sample sizes rather than the overall sample (Lakens, 2013). Cohen’s d values are represented by the color bars in Figures 2, 3. Cohen’s d of 0.2, 0.5, and 0.8 indicate small, medium, and large effects, respectively.

## Results

The primary results are summarized in Table 2. The MN and VAN comparisons between HCs and all ALS patients combined into one group were significant at *p* < 0.001 and remained so after family wise error correction. The same was true for between-patient group difference in the motor network (Table 3), but not in the VAN.

**TABLE 2:** Overview of the between-group differences in the DMN, MN, and VAN.

**Differences in the default mode network**				
	All ALS patients	ALSni	ALSci	ALS-FTD
HC	*P* < 0.005	*P* < 0.005		
ALSni				
ALSci				
ALS-FTD				
**Differences in the motor network**				
	All ALS patients	ALSni	ALSci	ALS-FTD
HC	*P* < 0.001*	*P* < 0.001*	*P* < 0.001*	*P* < 0.001*
ALSni			*P* < 0.001*	
ALSci				
ALS-FTD				
**Differences in the ventral attention network**				
	All ALS patients	ALSni	ALSci	ALS-FTD
HC	*P* < 0.001*	*P* < 0.005		
ALSni			*P* < 0.005	*P* < 0.005
ALSci				
ALS-FTD				

We have set differences that meet conservative significance thresholds in bold. Differences that survived family wise error correction for multiple comparisons. DMN, default mode network; MN, motor network; VAN, ventral attention network; ALS patients, amyotrophic lateral sclerosis; ALSni, ALS patients categorized as non-impaired; ALSci, ALS patients cognitively impaired; ALS-FTD, ALS patients with frontotemporal; dementia; HC, healthy control. *Indicates differences which survived FDR correction.

**TABLE 3:** Contrasts of the motor network, significant at *p*_*uncorr*_< 0.001.

**HC > ALS (All)**									
	**Peak level**				**MNI coordinates**			**Hemisphere**	**Structure**
		
**K**	***p*_*FDR*_**	**T**	**Cohen’s d**	***p*_*uncorr*_**	**x**	**y**	**z**		
**1,491**	**<.001**	**5.26**	**0.82**	**<.001**	**−42**	**−75**	**−12**	**L**	**Inferior occipital lobe**
**28**	**.002**	**4.21**	**0.66**	**<.001**	**−54**	**3**	**−15**	**L**	**Mid-temporal lobe**
**72**	**.002**	**4.12**	**0.64**	**<.001**	**−60**	**−12**	**−6**	**L**	**Mid-temporal lobe**
**23**	**.002**	**4.10**	**0.64**	**<.001**	**18**	**−72**	**39**	**R**	**Cuneus**
**33**	**.002**	**3.97**	**0.62**	**<.001**	**63**	**−12**	**18**	**R**	**Postcentral gyrus**

**HC > ALSni**									
	**Peak level**				**MNI coordinates**			**Hemisphere**	**Structure**
		
**K**	***p*_*FDR*_**	**T**	**Cohen’s d**	***p*_*uncorr*_**	**x**	**y**	**z**		

**74**	**.014**	**4.52**	**0.78**	**<.001**	**−42**	**−78**	**−12**	**L**	**Inferior occipital lobe**
**197**	**.014**	**4.03**	**0.69**	**<.001**	**39**	**−75**	**−18**	**R**	**Cerebellum Crus I**
**69**	**.014**	**3.99**	**0.69**	**<.001**	**−12**	**−75**	**−12**	**L**	**Cerebellum VI**
**59**	**.014**	**3.91**	**0.67**	**<.001**	**12**	**−66**	**−12**	**R**	**Cerebellum VI**

**HC > ALSci**									
	**Peak level**				**MNI coordinates**			**Hemisphere**	**Structure**
		
**K**	***p*_*FDR*_**	**T**	**Cohen’s d**	***p*_*uncorr*_**	**x**	**y**	**z**		

**575**	**<.001**	**5.27**	**1.12**	**<.001**	**45**	**−51**	**−18**	**R**	**Fusiform gyrus**
**748**	**<.001**	**5.16**	**1.10**	**<.001**	**−45**	**−69**	**−9**	**L**	**Inferior occipital lobe**
**ALSni > ALSci**									

	**Peak level**				**MNI coordinates**			**Hemisphere**	**Structure**
		
**K**	***p*_*FDR*_**	**T**	**Cohen’s d**	***p*_*uncorr*_**	**x**	**y**	**z**		

46	0.144	3.96	0.85	<.001	−45	−60	0	L	Mid-temporal lobe

**HC > ALS-FTD**									
	**Peak level**				**MNI coordinates**			**Hemisphere**	**Structure**
		
**K**	***p*_*FDR*_**	**T**	**Cohen’s d**	***p*_*uncorr*_**	**x**	**y**	**z**		

**79**	**.017**	**4.55**	**1.05**	**<.001**	**−60**	**−12**	**−3**	**L**	**Mid-temporal lobe**

*Bold indicates that conservative significance thresholds were met.*

**FIGURE 1:**
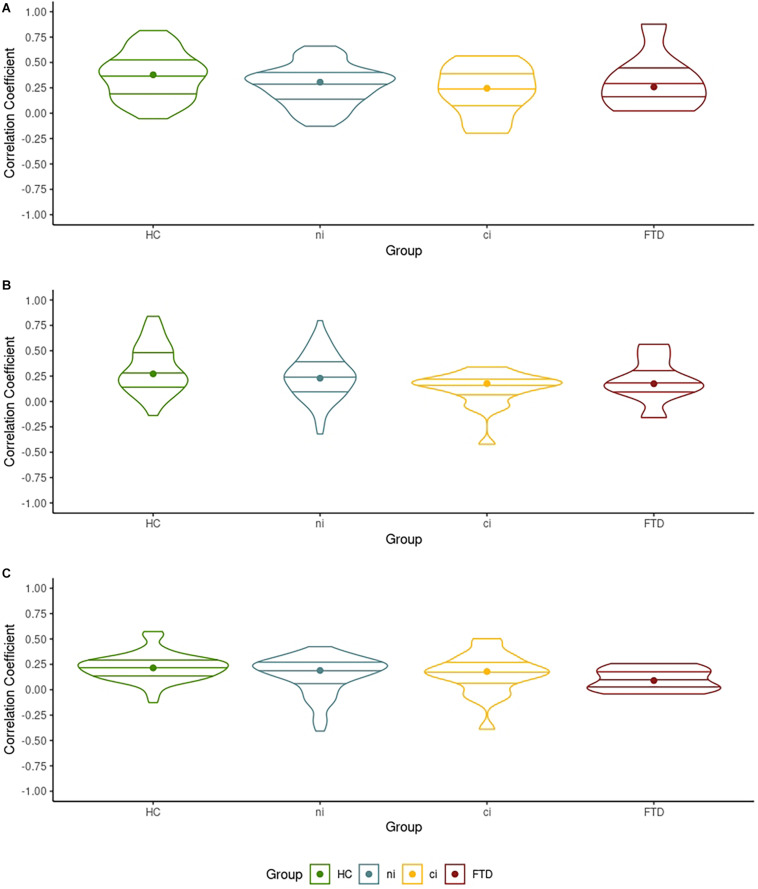
The distribution of the correlation coefficients between each network’s largest cluster and its seed voxel. **(A)** The distribution of the correlation coefficient in the left mid-frontal gyrus cluster in the default mode network (DMN) with the seed voxel. **(B)** The distribution of the correlation coefficient in the left fusiform gyrus cluster in the motor network (MN) with the seed voxel. **(C)** The distribution of the correlation coefficient of the left posterior-medial frontal gyrus cluster in the ventral attention network (VAN) with the seed voxel.

**FIGURE 2:**
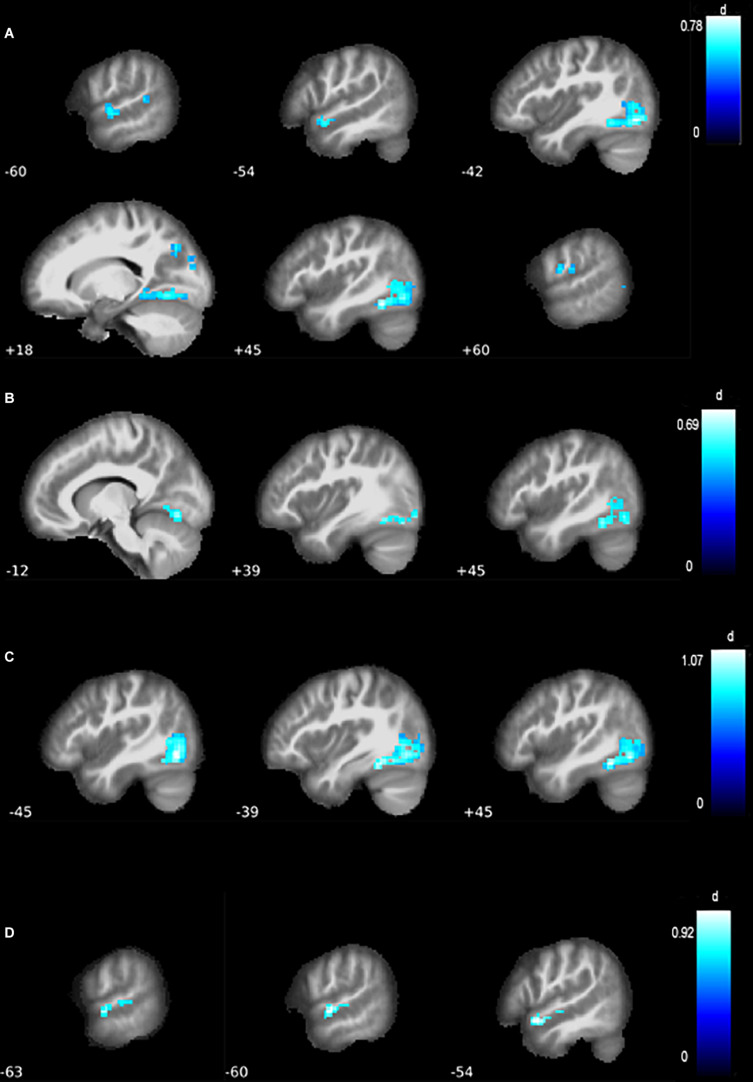
Differences in functional connectivity of the MN. **(A)** Compared with healthy controls (HCs), all amyotrophic lateral sclerosis (ALS) patients grouped together showed a contraction of the motor network, with reduced resting-state functional connectivity (RSFC) in the left inferior occipital lobe and mid-temporal lobe, as well as the right cuneus and postcentral gyrus. **(B)** Compared with HC, ALS patients without behavioral or cognitive impairments (ALSni patients) showed lower connectivity in the left inferior occipital lobe and bilateral cerebellum. **(C)** Compared with HC, cognitively impaired ALS patients (ALSci patients) showed reduced connectivity in the right fusiform gyrus and left inferior occipital lobe. **(D)** Compared with HC, ALS patients with additional frontotemporal dementia (ALS-FTD patients) patients showed reduced connectivity in the left mid-temporal lobe.

**FIGURE 3:**
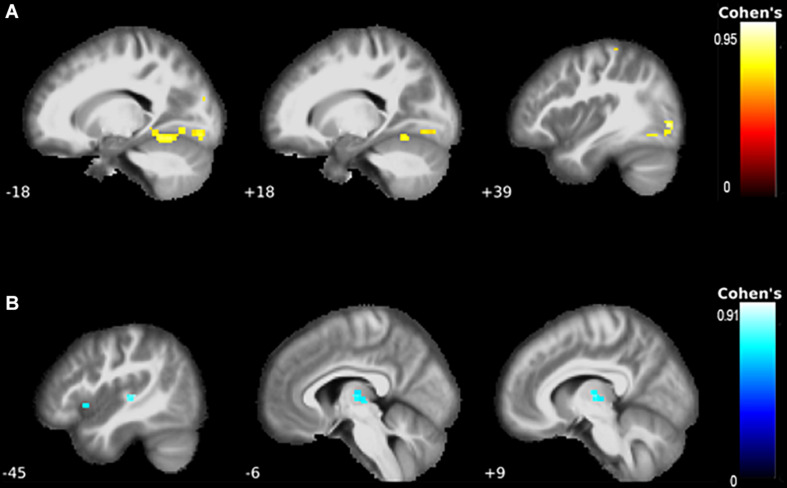
The correlations between functional connectivity and clinical presentation. **(A)** There were strong correlations between better-preserved motor function and the motor network. Specifically, the left fusiform gyrus, Rolandic operculum, mid-occipital lobe, lingual gyri, the right inferior occipital lobe, and postcentral gyrus showed higher connectivity with better motor function. **(B)** Worse shifting performance was associated with higher connectivity of the left thalamus and putamen.

A number of comparisons were only significant at the more liberal *P* < 0.005 (Table 2). Parametric mapping expects the RSFC values to be normally distributed. If they are non-normally distributed, the effects provided by the full-factorial ANOVA may be less reliable. Simultaneously, unequal group sizes may lead to heterogeneous distributions in the outcome, affecting the ANOVA’s reliability. To reinforce our effects’ reliability, we verified the normality and homogeneity of the RSFC values in each network by group (Figure 1) before reporting the primary analyses in detail (Figures 2, 3).

### Normality and Homogeneity of Each Network’s Functional Connectivity

Violin plots in Figure 1 show the dispersion of the correlation coefficient between each network’s largest cluster and its respective seed voxels, when comparing HCs with all ALS patients. Figure 1 informs us that the mean (indicated by the horizontal lines) and median correlation (indicated by the dots) across groups were similar, while densities varied across groups. In the DMN and VAN, all groups showed similarly sized interquartile ranges (indicated by the internal boxplots in Figures 1A,B), suggesting similar between-group variation in correlation coefficients. Shapiro–Wilk tests indicated normal distributions (*p* > 0.05) in 10 of the 12 groups in Figure 1. All 12 were homogeneous in their variances (Levene’s tests *p* > 0.05, Figure 1). Details can be found in our online Supplementary Material. Consequently, these data can be considered normally distributed and homogeneous in their variance (see Figure 1) despite the unequal group sizes, making them suitable for ANOVA.

### Differences in Resting-State Magnet Resonance Imaging Connectivity Networks Between Healthy Controls and Amyotrophic Lateral Sclerosis Patients

We report results significant at FDR-corrected *p* ≤ 0.001 and illustrate clusters of *k* ≥ 20 with Cohen’s d effect size (Figures 2, 3). Throughout, the blue color indicates negative associations, and the red color indicates positive associations.

At *p* < 0.001, there were no group differences in the DMN. Applying the more liberal *p* < 0.005, HCs showed increased connectivity of the left superior mid-frontal gyrus compared with ALS patients as a whole.

In the MN, ALS patients, as a whole, exhibited lower connectivity of the inferior occipital lobe (*p*_*FDR*_< 0.001, Cohen’s d = 0.82, Table 3, Figure 3A). Further differences emerged in the mid-temporal lobe, cuneus, and postcentral gyrus (*p*_*FDR*_ = 0.002, Cohen’s d = 0.62 to 0.66, Table 3). Lower connectivity in the ALS patients’ VAN’s supplementary motor area (*p* < 0.001) did not survive FDR correction despite a medium effect size (*p*_*FWE*_ = 0.094, Cohen’s d = 0.60, *k* = 24, *T* = 3.86, *x* = −3, *y* = 12, *z* = 54).

We now report differences between patient groups.

### ALSni Patients’ Functional Connectivity Networks

At *p*_*FDR*_ = 0.014, ALSni patients’ MN showed lower functional connectivity in the left inferior occipital lobe and the cerebellum (Table 3 and Figure 2B). ALSni patients’ VAN only exhibited lower functional connectivity compared with HC in the left supplemental motor area when applying the more liberal uncorrected *p* < 0.005 threshold.

### ALSci Patients’ Functional Connectivity Networks

In the MN, we observed a lower functional connectivity in the ALSci patients’ fusiform gyrus and inferior occipital lobe compared with the HC (*p*_*FDR*_< 0.001, Cohen’s d > 1, Table 3 and Figure 2C).

Differences in the VAN only emerged at the liberal uncorrected *P* < 0.005 threshold. ALSci patients exhibited lower functional connectivity in the mid-frontal gyrus, compared with ALSni patients.

### ALS-FTD Patients’ Functional Connectivity Networks

In the ALS-FTD patients’ MN, we observed lower functional connectivity than the HC for the mid-temporal lobe (*P*_*FDR*_ = 0.017, Table 3, Figure 2D).

Differences of the VAN only emerged at the liberal uncorrected *P* < 0.005 threshold. Compared with the ALSni patients, we observed a hypoconnectivity in the ALS-FTD patients’ left anterior cingulum.

### Correlations Between Clinical Presentation and Functional Connectivity Networks

For details refer to Table 4.

**TABLE 4:** Correlations between resting-state functional connectivity (RSFC) and clinical presentation.

**Positive correlations between ALSFRS-R and MN (*n* = 97)**									

	**Peak level**				**MNI coordinates**			**Hemisphere**	**Structure**
		
**K**	***p*_*FDR*_**	**T**	**Cohen’s d**	***p*_*uncorr*_**	**x**	**y**	**z**		
**145**	**.013**	**4.65**	**0.95**	**<.001**	**−33**	**−75**	**−12**	**L**	**Fusiform gyrus**
**26**	**.013**	**4.24**	**0.87**	**<.001**	**−42**	**−63**	**0**	**L**	**Mid-occipital lobe**
**181**	**.013**	**4.22**	**0.87**	**<.001**	**39**	**−81**	**−3**	**R**	**Inferior occipital lobe**
**40**	**.013**	**4.04**	**0.83**	**<.001**	**−27**	**−90**	**6**	**L**	**Mid-occipital lobe**
**37**	**.013**	**3.95**	**0.81**	**<.001**	**−60**	**3**	**6**	**L**	**Rolandic operculum**
**31**	**.013**	**3.94**	**0.81**	**<.001**	**60**	**−15**	**45**	**R**	**Postcentral gyrus**
**21**	**.013**	**3.86**	**0.79**	**<.001**	**−18**	**−87**	**−12**	**L**	**Lingual gyrus**

**Negative correlations between TMT B/A and VAN (*n* = 88)**									

	**Peak level**				**MNI coordinates**			**Hemisphere**	**Structure**
		
**K**	***p*_*FDR*_**	**T**	**Cohen’s d**	***p*_*uncorr*_**	**x**	**y**	**z**		

**21**	**.035**	**4.20**	0.91	**<.001**	**−9**	**−15**	**6**	**L**	**Thalamus**
**32**	**.035**	**3.99**	**0.86**	**<.001**	**−30**	**9**	**6**	**L**	**Putamen**

*Bold indicates that conservative significance thresholds were met.*

#### Standardized Montreal Cognitive Assessment Index and the Default Mode Network

There were no significant correlations between the MoCA index and the DMN.

#### ALSFRS-R and the Motor Network

A higher ALSFRS-R score—indicative of better-preserved motor function—correlated with increased connectivity of the left fusiform gyrus (*P*_*FDR*_ = 0.013, *k* = 145, *T* = 4.65, Figure 3A). Correlations with the mid-occipital lobes, inferior occipital lobe, Rolandic operculum, postcentral gyrus, and lingual gyrus did not survive FWE correction.

#### Standardized TMT Ratio and the Ventral Attention Network

Worse TMT ratio performance was associated with increased connectivity in the left thalamus and putamen (*P*_*FDR*_ = 0.038, Table 4, Figure 3B).

## Discussion

We applied a seed-based RSFC approach to investigate group differences between HC, ALSni, ALSci, and ALS-FTD patients in the default mode network, motor network, and ventral attention network. When applying conservative approaches to significance, we observed moderate to large differences between HC and patient groups in the motor network. The expected hypoconnectivity across all networks compared with controls was only present at a more liberal significance level. Applying this, we observed numerous differences in RSFC between ALS subgroups, but these did not follow any specific pattern. ALSci patients and ALS-FTD patients exhibited hypoconnectivity of the MN compared with ALSni. In the VAN, ALSci, and ALS-FTD patients showed hypoconnectivity compared with ALSni. None of these between-group effects outside the MN survived the FDR corrections. In the ALS patient group, we correlated the functional connectivity with three clinical parameters: motor function, global cognitive performance, and specifically shifting ability in the executive function. Worse performance on the executive function of shifting was associated with hyperconnectivity of the VAN even after the FDR correction. Conversely, better-preserved motor function was associated with hyperconnectivity of the motor network; this effect also survived the FDR correction. Throughout the recent ALS literature, the application of statistical thresholds and corrections has ranged heterogeneously from very liberal *P* < 0.05 without corrections to very conservative *P* < 0.001 with corrections (Mohammadi et al., 2009; Jelsone-Swain et al., 2010; Agosta et al., 2013; Zhou et al., 2013; Li et al., 2018). Consequently, we will discuss our findings at *P* < 0.001 and *P* < 0.005. None of our results ran in the opposite direction of our unidirectional hypotheses.

Conflicting findings regarding ALS patients’ divergent DMN connectivity have been reported previously: Agosta et al. (2013) observed a decrease in RSFC of the right orbitofrontal cortex and an increased RSFC of the precuneus in ALSni patients; Mohammadi et al. (2009) described a hypoconnectivity in the anterior and posterior cingulate cortex, medial prefrontal cortices, and inferior parietal cortices. Our findings did not support abnormal connectivity in the precuneus (Agosta et al., 2013), but contrary to the findings of Mohammadi et al. (2009), we found increased connectivity of the cingulate cortices in ALSni patients compared with ALS-FTD patients. Zhou et al. (2013) investigated the motor network connectivity of 12 ALS patients compared with healthy controls and found hypoconnectivity in the primary motor cortex and premotor cortex. Jelsone-Swain et al. (2010) also found hypoconnectivity of the motor network in ALS patients. Our results support such relative decreases between ALSci and HC as well as ALSni. Contrastingly, we observed hyperconnectivity in the left primary motor cortex of the ALS-FTD patients compared with ALSci. Li et al. (2018) applied a frequency—rather than seed-based approach to determine functional connectivity which yielded decreased functional connectivity of the lingual gyrus, postcentral gyrus, right superior temporal gyrus, left middle temporal gyrus, bilateral basal ganglia, and the ventrolateral prefrontal cortices. Our findings partially support those Li et al. (2018) and extend them to include different ALS patient groups. We observed decreased postcentral gyrus connectivity in the motor networks of our ALS patients and hypoconnectivity, not in the right, but in the left mid-temporal lobe of the ALS-FTD patients (Figure 2D). Loewe et al. (2017) reported reduced RSFC in the occipital lobes of ALS patients. Our results support and expand this finding: the reduced connectivity occurred specifically when comparing controls with ALSni and ALSci patients. We did not find changes in the ventrolateral prefrontal cortices, even though increased RSFC of the VAN correlated with impaired executive functions (Figure 3B).

Two theoretical explanations are consistent with our findings: compensatory functional activity and the spectral nature of the ALS-FTD proteinopathies. These explanations are not mutually exclusive.

Roosendaal et al. (2010) hypothesized that subclinical structural damage in early disease is compensated for by functional activity, so clinical impairment only becomes obvious after compensatory mechanisms are exhausted. Our present and previous observations of this sample are consistent with this hypothesis: cortical thinning occurred in the motor cortex, right-sided frontotemporal cortices, and the right inferior precuneus, and was associated with cognitive impairment in a subset of this sample (Schuster et al., 2013, 2014). In a smaller subset, TDP-43 was also associated with cognitive impairment (Prudlo et al., 2016). Heimrath et al. (2014) reported that cognitive deficits were associated with hyperconnectivity in parahippocampal and parietal areas of the DMN in nine ALS patients. Moreover, faster disease progression has been related to motor network hyperconnectivity (Verstraete et al., 2010). The presently reported RSFC patterns in ALS-FTD support the notion of compensatory hyperconnectivity in the premotor cortex of the MN and the putamen and insula lobe. Simultaneously, our ALS-FTD patients had better-preserved motor function and shorter disease duration than the ALSni and ALSci groups. Putaminal lesions have been associated with executive dysfunction, while insular damage has been associated with reduced emotional awareness (Sefcsik et al., 2009; Jones et al., 2010), competencies that are impaired in ALS-FTD. Regarding the overall sample, we observed that better-preserved motor function was associated with hyperconnectivity of the MN, while worse cognitive functions were associated with hyperconnectivity. An association between hyperconnectivity of the MN and motor function has previously been documented using EEG (Dukic et al., 2019). Combined, our findings support the hypothesis that hyperconnectivity occurred in areas that were structurally damaged and with support functions vulnerable to disease-specific impairment.

**FIGURE 4:**
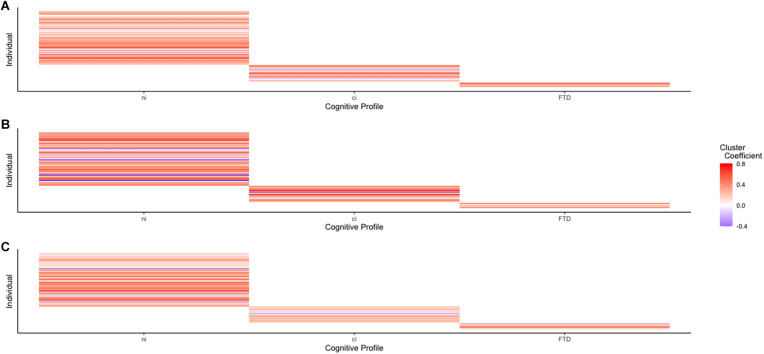
Connectivity between each network’s largest cluster and its seed voxels shows no discernible pattern, supporting the idea of an ALS-FTD spectrum. **(A)** Cluster connectivity of the DMN across the ALS-FTD spectrum. **(B)** Cluster connectivity of the VAN across the ALS-FTD spectrum. **(C)** Cluster connectivity of the MN across the ALS-FTD spectrum.

ALS-FTD is increasingly recognized as a spectrum of cognitive–behavioral impairments (Murphy et al., 2007), underlain by a spectrum of proteinopathies (Geser et al., 2009; Mackenzie et al., 2010) and driven by an etiologically diverse molecular genetic spectrum (Van Langenhove et al., 2012; Müller et al., 2018). These spectra present alongside a possible morphological continuum of reduced cortical thickness, ranging from ALSni to ALS-FTD (Rohrer et al., 2009, 2010; Schuster et al., 2014; Agosta et al., 2016). Shen et al. (2018) investigated whether the cognitive profiles of ALS showed distinct perfusion patterns and found that hypoperfusion often occurred alongside atrophy. Atrophy and hypoperfusion worsened gradually between ALSni, ALSci, and ALS-FTD patients (Shen et al., 2018), suggesting that hypoperfusion occurs as a continuum. In our study, liberally determined differences in RSFC networks signified hypoperfusion between ALSni and ALSci patients, alongside hypo- as well as hyperperfusion between ALS-FTD and ALSni and ALSci patients. This suggests that RSFC is of a spectral nature: if there were a continuum with ALSni and ALS-FTD at its opposite ends, Figure 4 would contain a discernible pattern (and Figure 1 would contain progressive changes in violin shape). Instead, we see distinctive profiles between groups.

Ours was a prospective study with homogenized operating procedures and identical MRI acquisition protocols at both sites, including phantom tests [following the guidelines of the American College of Radiology (American College of Radiology, 2018)]. Analyses incorporated age, sex, location, and verbal IQ to account for their effects on connectivity and cognition, and we ascertained the normality and homogeneity of our data to document the reliability of our main results in light of our unequal group sizes (Figure 1).

Our study has three major limitations. First, uneven group sizes are undesirable even when normality and homogeneity of variance are present, and even when they reflect the natural dispersion of cognitive impairment, as is the case here. However, the proportion of the ALS-FTD group within the broader ALS cohort in our study (8.2%) would, in fact, reflect the proportional numbers expected among ALS patients in general. Second, we did not include a measure of social cognition, so we could not investigate the previously proposed association of cingulate connectivity and this domain (Trojsi et al., 2017). Third, our findings varied greatly depending on the significance levels. This may be rooted in the very small ALS-FTD group and relates back to the first limitation. Fourth, future work might also include behaviorally impaired profiles of the ALS-FTD spectrum. These challenges leave room for future methodological expansions of the present work.

In conclusion, our findings support distinct rs-fMRI network connectivity in cognitive subgroups of ALS. This encourages the notion that ALS is a spectrum disorder with ALSni, ALSci, and ALS-FTD presenting distinctly, without progression between them. Our observation that worse attentional shifting correlated directly with VAN connectivity reinforces the idea that increased connectivity is a support mechanism.

## Data Availability Statement

The datasets presented in this article are not readily available because the MRI data cannot be shared publicly due to ethical constraints. Requests to access these datasets should be directed to JP, johannes.prudlo@med.uni-rostock.de.

## Ethics Statement

The studies involving human participants were reviewed and approved by the Ethics Committee of the University of Rostock, and of the University of Magdeburg. The patients/participants provided their written informed consent to participate in this study.

## Author Contributions

AGMT and MD developed the methodology, performed the statistical analysis, interpreted the results, wrote the original draft (Methodology, Results, and Discussion). CB conceptualized the study, conducted the statistical analyses, and wrote the original draft (Introduction). EK and JM also conceptualized the study, developed the neuropsychological battery, collected the neuropsychological data, and reviewed the manuscript. JK developed the imaging methodology, collected the imaging data, and reviewed the manuscript. SV also conceptualized the study, collected the neurological data, and reviewed the manuscript. ST also conceptualized the study, performed the statistical analysis, interpreted the results, and reviewed the manuscript. JP also conceptualized the study, collected the neurological data, acquired the grant, and reviewed the manuscript. All authors contributed to the article and approved the submitted version.

## Conflict of Interest

ST participated in scientific advisory boards of Roche Pharma AG, Biogen, Grifols SA, Dr Willmar Schwabe GmbH & Co. KG, and MSD, and received lecture fees from Roche Pharma AG and MSD. The remaining authors declare that the research was conducted in the absence of any commercial or financial relationships that could be construed as a potential conflict of interest.
