# Supplementary material for: Cognitive Profiles of Amyotrophic Lateral Sclerosis Differ in Resting-State Functional Connectivity: An fMRI Study
Source: Front Neurosci. 2021 Jun 23;15:682100. doi: 10.3389/fnins.2021.682100 (PMC8261303; doi:10.3389/fnins.2021.682100)

"Cognitive profiles of amyotrophic lateral sclerosis differ in resting-state functional connectivity: an fMRI study"

This JASP-based HTML results file presents supplemental analyses for the manuscript "Cognitive profiles of amyotrophic lateral sclerosis differ in resting-state functional connectivity: an fMRI studyCognitive profiles of amyotrophic lateral sclerosis differ in resting-state functional connectivity: an fMRI study" by Temp et al., submitted to *Frontiers in Neuroscience*.

These supplemental analyses demonstrate that despite the differences in group size, our data met the assumptions of normality and homogeneity, making them suitable for ANOVA.

Variable Dictionary

1. DMNclustercoefficient: Pearson's correlation coefficient in the largest cluster of the DMN
2. VANclustercoefficient: Pearson's correlation coefficient in the largest cluster of the VAN
3. Mncustercoefficient: Pearson's correlation coefficient in the largest cluster of the MN
4. Group: distinction according to the most recent Strong criteria, with the levels:
    a. HC: healthy controls (n=69)
    b. FTD: ALS-FTD patients (n=8)
    c. ci: ALS patients with cognitive impairment (n=21)
    d. ni: ALS patients without cognitive or behavioural impairment (n=68)

Normality of Raw Scores

The below table shows the number of cases in each group, the measures of dispersion and the results of a Shapiro-Wilk test. These data accompany Figure 1 in the manuscript.

Descriptive Statistics

	DMNclustercoefficient				VANclustercoefficient				Mncustercoefficient			
	FTD	HC	ci	ni	FTD	HC	ci	ni	FTD	HC	ci	ni
Valid	8	69	21	68	8	69	21	68	8	69	21	68
Missing	0	0	0	0	0	0	0	0	0	0	0	0
Mean	0.304	0.366	0.226	0.274	0.103	0.222	0.159	0.147	0.204	0.319	0.126	0.245
Std. Deviation	0.277	0.223	0.239	0.195	0.111	0.131	0.200	0.183	0.217	0.234	0.167	0.221
Shapiro-Wilk	0.885	0.973	0.937	0.977	0.904	0.970	0.951	0.903	0.961	0.966	0.841	0.994
P-value of Shapiro-Wilk	0.211	0.142	0.191	0.249	0.313	0.101	0.362	< .001	0.819	0.060	0.003	0.985
Minimum	0.023	−0.054	−0.198	−0.128	−0.041	−0.126	−0.389	−0.407	−0.159	−0.138	−0.421	−0.320
Maximum	0.877	0.815	0.565	0.661	0.258	0.574	0.504	0.425	0.564	0.839	0.339	0.797

As noted, the vast majority of our subgroups' cluster coefficients were normally distributed across networks. Notable exceptions from the table above: the VAN coefficients of ALSni patients (*p*<.001) and the MN coefficients of healthy controls (*p*=.003). However, ANOVA are robust to the violation of normality; and we applied Fisher's Z transformation, as is customary.

DMN: Homogeneity of Variances

We conducted this ANOVA solely to demonstrate that it met the necessary statistical assumptions.

ANOVA - DMNclustercoefficient

Cases	Sum of Squares	df	Mean Square	F	p	ω^2
Group	0.451	3	0.150	3.196	0.025	0.038
Residuals	7.622	162	0.047			

Note. Type III Sum of Squares

Assumption Checks

Levene's test below is non-significant, indicating that the between-group variance is homogeneous despite the unequal group sizes.

Test for Equality of Variances (Levene's)

F	df1	df2	p
0.829	3.000	162.000	0.480

Q-Q Plot

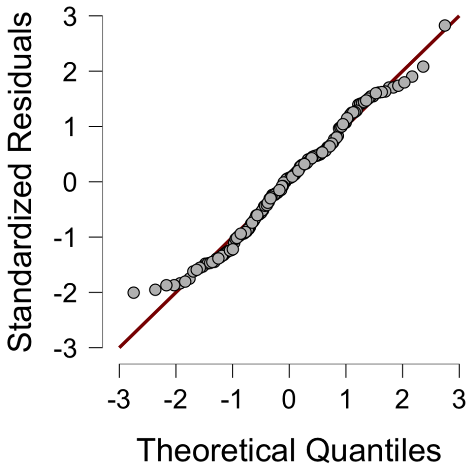

The QQ plot above shows signs of negligible deviations from normality in the ANOVA's residuals. As indicated in the manuscript, Fisher's Z transformation was applied to the raw scores to address the matter.

VAN: Homogeneity of Variances

ANOVA - VANclustercoefficient

Cases	Sum of Squares	df	Mean Square	F	p	ω^2
Group	0.255	3	0.085	3.206	0.025	0.038
Residuals	4.298	162	0.027			

Note. Type III Sum of Squares

Assumption Checks

Levene's test below is non-significant, indicating that the between-group variance is homogeneous despite the unequal group sizes.

Test for Equality of Variances (Levene's)

F	df1	df2	p
2.234	3.000	162.000	0.086

Q-Q Plot

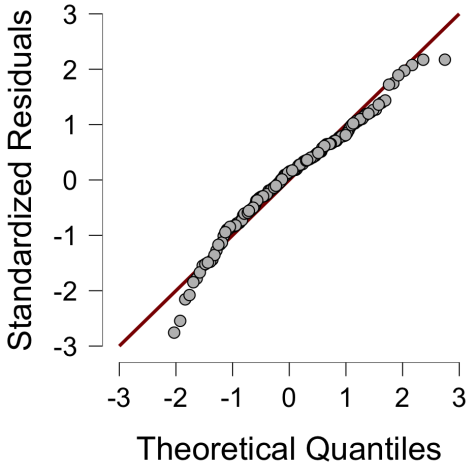

MN: Homogeneity of Variances

ANOVA - Mncustercoefficient

Cases	Sum of Squares	df	Mean Square	F	p	ω^2
Group	0.658	3	0.219	4.515	0.005	0.060
Residuals	7.867	162	0.049			

Note. Type III Sum of Squares

Assumption Checks

Levene's test below is non-significant, indicating that the between-group variance is homogeneous despite the unequal group sizes.

Test for Equality of Variances (Levene's)

F	df1	df2	p
1.826	3.000	162.000	0.145

Q-Q Plot

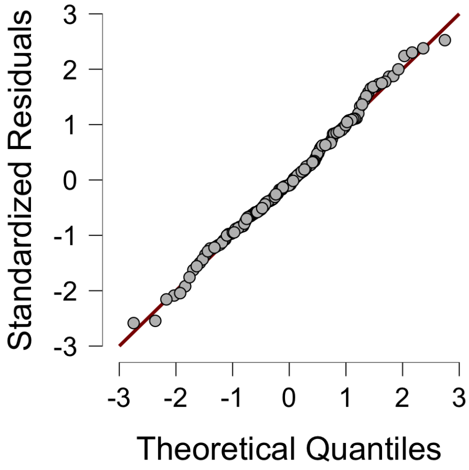

Supplement: Supplementary file 1 [file Data_Sheet_1.PDF]
